# Acceptability of a sitting reduction intervention for older adults with obesity

**DOI:** 10.1186/s12889-018-5616-1

**Published:** 2018-06-07

**Authors:** Theresa E. Matson, Anne D. Renz, Michelle L. Takemoto, Jennifer B. McClure, Dori E. Rosenberg

**Affiliations:** 1 Kaiser Permanente Washington Health Research Institute, Seattle, WA USA; 2 Department of Family Medicine and Public Health, University of California San Diego, La Jolla, CA USA

**Keywords:** Sedentary behavior, Sitting reduction, Older adults, Elderly, Chronic conditions, Obesity, Qualitative, Technology

## Abstract

**Background:**

Older adults spend more time sitting than any other age group, contributing to poor health outcomes. Effective behavioral interventions are needed to encourage less sitting among older adults, specifically those with obesity, but these programs must be acceptable to the target population. We explored participant acceptance of a theory-based and technology-enhanced sitting reduction intervention designed for older adults (I-STAND).

**Methods:**

The 12-week I-STAND intervention consisted of 6 health coaching contacts, a study workbook, a Jawbone UP band to remind participants to take breaks from sitting, and feedback on sitting behaviors (generated from wearing an activPAL device for 7 days at the beginning and mid-point of the study). Semi-structured interviews were conducted with 22 participants after they completed the intervention. Interview transcripts were iteratively coded by a team, and thematic analysis was used to identify and refine emerging themes.

**Results:**

Overall, participants were satisfied with the I-STAND intervention, thought the sedentary behavior goals of the intervention were easy to incorporate, and found the technologies to be helpful additions to (but not substitutes for) health coaching. Barriers to standing more included poor health, ingrained sedentary habits, lack of motivation to change sedentary behavior, and social norms that dictate when it is appropriate to sit/stand. Facilitators to standing more included increased awareness of sitting, a sense of accountability, daily activities that involved standing, social support, and changing ways of interacting in the home environment. Participants reported that the intervention improved physical health, increased energy, increased readiness to engage in physical activity, improved mood, and reduced stress.

**Conclusions:**

The technology-enhanced sedentary behavior reduction intervention was acceptable, easy to incorporate, and had a positive perceived health impact on older adults with obesity.

**Trial registration:**

The I-STAND study was registered at clinicaltrials.gov (ID: NCT02692560) February 2016.

**Electronic supplementary material:**

The online version of this article (10.1186/s12889-018-5616-1) contains supplementary material, which is available to authorized users.

## Background

Adults over the age of 65 spend more time sitting than any other age group [1, 2] and more frequently experience chronic health problems associated with obesity [3, 4]. Older adults with a body mass index (BMI) greater than 30 kg/m^2^ spend over 10–11 h per day sitting, putting them at particular risk for chronic diseases [1].

Sedentary behavior includes time spent exerting minimal energy (≤1.5 METs) while seated or lying down [5]. Sitting at the computer and watching TV are common sedentary behaviors, while standing at the counter to cook is non-sedentary. Long periods of sitting are associated with less successful aging as exemplified by the presence of chronic diseases, functional impairment, and psychological or social distress [6].

Greater engagement in physical activity cannot compensate for long periods of sitting [2, 7]. Even though standing requires low energy expenditure, it engages large muscles of the lower body, which can prevent health consequences associated with metabolic problems [8]. Considering that 70% of Americans do not meet recommendations for physical activity [9] and 60% of waking hours are spent sedentary [1], interventions specifically targeting sitting reduction are warranted.

Several small pilot studies indicate that reducing sitting time among older adults is feasible [10–15]. In a feasibility study focused on obese older adults, we found reductions of 30 min per day [11] comparable to other pilot studies in non-obese older adults [10, 12–16]. Effective behavioral interventions are needed to encourage less sitting among older adults, particularly older obese adults, but these programs must also be acceptable to this target population to encourage long-term adoption.

Sedentary behavior interventions for older adults must demonstrate the importance of reducing sedentary behavior and counteract perceived barriers to reducing sitting. Qualitative findings from our feasibility study suggest that older adults are unaware of how long they are sedentary and need to learn specific strategies, which are quite different from those employed in physical activity promotion interventions, to interrupt a strongly ingrained habit of sitting [17, 18]. Older adults have also reported anticipated fatigue or pain and the perception that work and leisure activities must be done sitting as barriers to standing more [19]. However, sedentary behavior interventions for older adults may be able to address these barriers by setting reminders to take periodic breaks from sitting (e.g., at every commercial) [20], by setting small goals that promote self-efficacy to stand [21], and by making environmental changes that promote standing (e.g., standing desk) [22]. No study has assessed older adults’ interest and willingness to incorporate these strategies into a sedentary behavior intervention and the barriers and facilitators they experience as a result. If sedentary behavior reduction is acceptable to a high-risk population such as older adults with obesity, it could provide alternative public health approaches.

### Study purpose

We developed a 12-week intervention called I-STAND that uses a tailored approach to reduce sitting time and to increase the number of breaks from sitting among older adults with obesity who spend more than 8 h per day sitting. I-STAND was based on a pre-post feasibility study that reduced sitting time by 30 min per day [11, 18]. Interview data from the feasibility study were used to refine the I-STAND intervention and incorporate new strategies, such as wrist-worn technology to prompt breaks from sitting, that address previously identified barriers [18]. In the current study, we analyzed qualitative in-depth interviews administered after the I-STAND intervention to understand acceptability of the program, to expand our understanding of barriers and facilitators to behavior change over a prolonged period, and to explore potential health impacts. This study also assessed the acceptability of the Jawbone UP band, interest in other types of technology, and perceptions of using technology-based interventions to inform future research in older adult populations.

## Methods

### Setting

The study was conducted at Kaiser Permanente Washington Health Research Institute (formerly known as Group Health Research Institute). All research activities were reviewed and approved by the Kaiser Permanente Washington Institutional Review Board. The study was conducted between February 2016 and February 2017. Additional detail on the parent trial can be found at ClinicalTrials.gov (NCT02692560).

### Study sample

Older adults between the ages of 60 and 89 years were randomly recruited into the I-STAND study from Kaiser Permanente Washington (KPWA) membership panels. Electronic medical records and telephone screens completed by a research specialist were used to identify patients who met the following inclusion criteria: over age 60, BMI 30–50 kg/m^2^, not residing in long-term care or skilled nursing, no diagnosis of dementia, and no serious mental or a potentially terminal illness (e.g., cancer, heart failure). Participants were excluded if they reported being unable to stand, were not able to walk one block, were participating in another intervention study at the time of recruitment, reported sitting time of less than 7 h per day, could not communicate by phone, or could not speak and read English. Informed consent was provided verbally during the telephone screen and in writing at the baseline visit. A total of 60 participants met eligibility criteria and consented to participate in the intervention. Of these participants, 29 were randomized to the I-STAND intervention and 31 were randomized to a Healthy Living control arm. A convenience sample of I-STAND intervention participants completed semi-structured exit interviews following study completion.

### Intervention

#### I-STAND

Participants in the experimental arm received the I-STAND intervention. The intervention was based on behavioral theories including social cognitive theory [21], the ecological model [22], and habit formation theory [20]. I-STAND combined the aforementioned behavioral theories into an approach focusing on using personalized reminder strategies to increase awareness of overall sitting time and encourage participants to make simple changes that promote self-efficacy and reduce sedentary behavior. Reminder strategies included outward cues in the environment (e.g., timers or visual cue cards placed on chairs to prompt a break from sitting), inner cues that involve being mindful of bodily discomfort when sitting for long periods of time, and habit-associated cues that made use of daily behaviors to signal reminders to stand (e.g., standing for 5 min while drinking coffee or talking on the phone).

During the first two weeks, intervention participants met with their health coach in-person twice for one hour. In the following weeks, they received 4 biweekly phone calls. Participants were given a study workbook that included educational information, action planning, and goal-tracking worksheets. Participants were also given a Jawbone UP band, which is a wrist-worn device programmed to gently vibrate every 15 min of inactivity; this served as an outward reminder strategy for taking breaks from sitting. Data from a thigh-worn, accelerometer-based device (activPAL, Glasgow, Scotland) were used to generate feedback charts for I-STAND participants after baseline, Week 1, and Week 6 (study mid-point) that showed number of hours spent sitting, number of 30-min periods of prolonged sitting, number of breaks from sitting, and number of steps taken. During each session, the participant and health coach reviewed and set personalized sedentary behavior goals, problem-solved barriers to goal attainment, and discussed personalized reminder strategies.

### Procedures & Data Collection

Procedures for the trial are described in detail elsewhere [23]. For this qualitative study, exit interviews were conducted with I-STAND participants within 10 days of completing final measurements. Interviews were conducted by a team member who did not have any prior contact with participants during the study to reduce response bias. The interviewer (T.M.) followed a semi-structured interview guide with open-ended questions and follow-up prompts (see Additional file 1). Questions asked about participants’ overall impressions, barriers and facilitators to changing sedentary behavior, and perceived impact of the intervention on physical and mental health. Questions also asked about the Jawbone UP band, each specific intervention component (e.g., health coach sessions, feedback charts, reminder strategies), and interest in using other technologies such as e-mail or web to deliver the program. Interviews averaged 47 min (range = 25–80 min). Each call was audio-recorded with consent and then transcribed for analysis. Participants were compensated for study participation, but not compensated for completing the qualitative interview.

### Data analysis

We conducted inductive and deductive thematic analysis [24]. A coding team of three individuals (A.R., T.M., and D.R.) read all transcripts and developed a codebook which was iteratively refined throughout the coding process. Each transcript was coded independently by two coders using the most current codebook. Any differences were resolved through consensus. Final codes were applied by one coder who ensured that transcripts coded earlier were reconciled with the most current codebook. Coders had relevant backgrounds (A.R., T.M.: public health; D.R:. public health, clinical psychology) and all had training and coursework in qualitative methods and analysis. Final codes were entered into ATLAS.ti 7.5. Repeated rounds of reading and categorizing output for each code helped establish themes and refine interpretation. Coding occurred over the course of several months and saturation was reached when no new codes were generated after a final review of the transcripts. Direct quotes from participants were selected to illustrate themes. Themes were summarized into three domains: acceptability of I-STAND program components and goals, barriers and facilitators to achieving sitting reduction goals, and perceived health impact of reducing sedentary time.

## Results

Based on logistics, 23 out of the 29 I-STAND intervention participants were invited to complete qualitative interviews. Six participants were not contacted because health coaches reported they were unavailable, uninterested, or inappropriate for interview. Of the I-STAND participants contacted, only one participant refused a formal interview citing lack of time. A total of 22 interviews were conducted, and saturation was reached. Interview participants (*n* = 22; 100% white, 63.6% female, 77.3% had a college degree or higher) had an average age of 69 years with a mean BMI of 35.8 kg/m^2^. Participant characteristics are described in Table 1. Table 2 presents an overview of themes. Additional quotes are provided in a supplementary table (see Additional file 2).

**Table 1:** Baseline characteristics of I-STAND exit interview participants

Characteristic	n (%)
N	22
Sex (female)	14 (63.6%)
Age (years), mean (SD)	69.2 (4.9)
Race (White)	22 (100%)
Highest level of education	
Some college	5 (22.7%)
College graduate (4-year degree)	12 (54.6%)
Graduate or professional degree	5 (22.7%)
Employment	
Fulltime	2 (9.1%)
Part time	4 (18.2%)
Retired	16 (72.7%)
Diabetes	7 (31.8%)
Hypertension	15 (68.2%)
Body mass index (kg/m^2^)	35.8 (6.2)

**Table 2:** Overview of themes

Domain	Theme	Examples
Acceptability of sedentary behavior reduction intervention	Overall satisfaction	Health coaches, delivery, timeline, goal-setting
	Technology	activPAL, Jawbone UP, interest in smart phone application or online communication
	Ease and effectiveness	Easy to incorporate into daily routine, high self-efficacy
Barriers to reducing sedentary behavior	Poor health	Back pain, knee pain, foot pain, fatigue, sickness
	Ingrained sedentary habits	TV watching, reading, crafting, playing games, socializing, eating, talking on the phone
	Lack of motivation and other priorities	“Sitting is a reward” mindset, favorite activities involve sitting, caretaking, commuting
	Social norms	Business meetings, restaurants, theaters, bus, public outings
Facilitators to reducing sedentary behavior	Increased awareness	activPAL/feedback charts, Jawbone UP, outward reminders, habit-associated reminders, inner reminders
	Accountability	Health coaches, activPAL/feedback charts, goal-setting
	Daily activities	Being able to accomplish chores, caretaking, yardwork, walking
	Social support and norms	Family, friends, co-workers, health coach, non-judgment in public
	Changing ways of interacting with home environment	Leaning against tall chairs or handrails, doing activities at standing height counters, moving items further away from bed or couch
Perceived health impact	Physical benefits	Weight loss, lower blood pressure, resolution of sciatica, less pain, reduced swelling in ankles, core muscle strength, increased flexibility, ability to get up and move, increased stamina
	Increased energy	Less sleepy/sluggish/lethargic, increased stamina to accomplish activities, “good” tired at end of the day
	Increased interest in physical activity	Feels mentally and physically prepared to start or do more physical activity, has already incorporated more physical activity into routine (e.g., with stepping goals)
	Improved mood	Happier, doing more rewarding activities, feelings of accomplishment, reframe negative mindset about taking breaks from sitting
	Reduced stress	Able to accomplish more in the day, anticipated health benefits from reduced sedentary behavior

### Acceptability

Overall, participants were highly satisfied with the I-STAND intervention and felt the goals of the intervention (i.e., reducing sitting time by standing for longer periods and taking more frequent breaks from sitting) were appropriate and relevant to their needs. One participant described it as “*a wonderful experience, and it changed my life*” (P1042). The same participant elaborated,

“*I’m getting older and trying to do what I can to help my health, and the timing was absolutely perfect for me to participate in this study. My brain and my body were both ready for something too. I’ve not been perfect about doing everything within the study, and my numbers may not be good, but my lifestyle has changed pretty dramatically. I’m able to stand on my feet for long periods of time now that I couldn’t before.*” (P1042)

All participants expressed satisfaction with their health coaches. Specifically, participants appreciated the role health coaches played in keeping them accountable to their goals and tailoring the intervention to meet their needs. “*I think having a* [health coach who] *helped me with the goal setting was incredibly valuable. Not just the goal setting but the follow up of the goal setting and the future planning for at the end of the study—what am I gonna do then*” (P1078). Most participants also thought the length (12 weeks) and format (2 in-person visits with 4 phone calls and mailed material) of the intervention were appropriate, although some wanted the intervention to be longer.

#### Technology

Participants expressed a range of opinions regarding technical components of the intervention. Participants enjoyed getting feedback charts that tracked sedentary behavior, but a few participants did not like wearing the activPAL. Reasons for disliking the activPAL included: skin irritation and break outs where worn, difficulty putting it on, and pain when removing it. Even so, most thought the discomfort was minor and that it was worthwhile to wear the activPAL because they liked the feedback charts summarizing their data. “*They were probably one of the best resources to monitor my daily movements and I thought they were fabulous*” (P1188).

Most participants thought the wrist-worn Jawbone UP band was a useful tool for prompting reminders to stand. P1538 said, “*The Jawbone was probably the best reminder of everything during the full course of the program*.” P1255 added, “*The little buzz it would give you is helpful in a way that, say, just trying to keep track of time on your own is not.*” However, there were several for whom it was not ideal. “*I did not like the Jawbone. I stopped using that because it was really irritating. Even when I thought I had gotten up and moved around, it was still going off*” (P1163). Chief complaints regarding the Jawbone UP band included: poor sensitivity to movement, programmed to go off too frequently (after 15 min of sedentary time), uncomfortable to wear around the wrist, and difficult to put on or connect to the charger. Participants tended to have strong positive or strong negative opinions about the Jawbone UP band, but the majority favored its use. Even participants who did not like using the Jawbone UP band were not inherently opposed to the idea of using some form of wearable technology as a reminder device if their usability concerns were addressed. P1110 said, “*I would have very willingly wore a wristband if it was more easy, you found one that is not dark, and one that’s easy to see, and a wristband that the patient or a participant can manipulate.*”

Participants were also asked about their comfort with other technologies to communicate with their health coach (e.g. through e-mail, web platform, or smartphone app), and if they would be interested in getting additional feedback on their activity level (e.g. real-time readouts of sitting time, step count, etc.) that would require use of technology to collect. Although over half of participants felt comfortable using technological devices, most indicated they were not interested in using it as the primary means of communicating with their health coach. “*So it’s not that I’m not capable of using a web-based program, but I don’t think it would have the same—it just seems really impersonal*” (P 1038). However, participants were interested in using technology if it meant they would receive daily or more detailed feedback in addition to phone or in-person interactions with a health coach.

#### Ease and effectiveness

Almost all participants felt they reduced their sitting time, and many participants attributed this improvement to the ease of incorporating the goals of the intervention into their daily lives.

“*I surprised myself how absolutely sedentary I was, and then how I was able to make some changes that actually didn’t feel really difficult. I was thinking, ‘Oh, my god, I’m gonna have to stand here,’ but it wasn’t like that. Anyway, it was a positive experience.*” (P1697)

P1879 elaborated, “*You don’t need special equipment, you don’t have to have the right kind of clothes, you don’t have to go anyplace, it doesn’t cost you a gym membership. You just have to stand up.*” Moreover, most participants felt confident in their ability to maintain sedentary behavior changes and motivated to further reduce sedentary behavior.

### Barriers

Although participants found the intervention acceptable and easy to incorporate into their daily lives, participants also identified several barriers to changing sedentary behavior.

#### Poor health

New and pre-existing health problems made it difficult for some participants to increase their standing time and take more frequent breaks from sitting. Specifically, participants reported that a change in medication, repercussions from a recent fall, and sickness and fatigue were barriers to reducing sitting time. As P1169 describes, “*if I’m feeling tired it’s much harder for me to get up and move around.*” Pre-existing conditions such as prior surgery, arthritis, or obesity also made standing more difficult. “*Well, I’m a heavy woman, and so it’s hard to stand in one place because it just—it makes my knees hurt. It’s just uncomfortable*” (P1188). However, participants with pre-existing conditions overwhelmingly found they were able to reduce pain associated with prolonged standing through walking, taking short but frequent breaks from sitting, or leaning against something for support.

“*I think because I’m overweight with a big belly, if I’m not moving, it puts a little stress on my back and makes my back sore, so I’m trying to find more opportunities for standing. One of the things I did is take one of my kitchen chairs and put it in the living room so I can stand while I’m watching TV and just have something to lean on a little bit, but I’m still standing.*” (P1538)

Participants reported that pain associated with pre-existing conditions lessened as they decreased their sitting.

#### Ingrained sedentary habits

Participants frequently described difficulty breaking the habit of sitting. After years of associating daily activities such as eating, reading, socializing, TV viewing, etc. with sitting, it had become an ingrained habit. P1053 describes this habit as one of the biggest barriers to reducing sitting:

“*The habit of always sitting no matter where I was or what I was doing is, for the most part, is I’d just gotten into the habit of sitting, whether it was watching TV, reading, or whatever. And that was about it. That was about the worst. I was trying to overcome that.*”(P1053)

Participants also emphasized that, for most of their life, the decision to sit was made unconsciously. “*So you know, a lot of your activity is sitting, so it’s a challenge. You have to kind of think about it, which I didn’t really do much before; thinking about whether I was standing or sitting*” (P1360).

#### Lack of motivation and competing priorities

Some participants cited a lack of motivation and strong preference for sitting as a barrier to standing: “*creature comforts become more important as you get older*” (P1042). Others felt that sitting was deserved in old age:

“*I was looking forward to this aspect of retirement that I, you know—I’d be able to indulge some of my other passions like reading, studying, intellectual pursuits… And I kind of resented this idea of: What do you mean, I can’t sit down?*” (P1086)

For most, the preference for sitting was motivated by sedentary activities. As P1255 stated, “*We watch TV in the evening, and my favorite hobbies during the day are reading and knitting, both of which are sedentary, generally sedentary activities*” (P1255). Others were motivated by concerns and responsibilities that distracted from sedentary behavior or responsibilities that were more important. P1133 described, “*I’ve got caretaking responsibilities, and they come before standing up if I’m in the middle of doing something that requires me to sit down with the baby*” (P1133).

#### Perceived social norms

Some participants perceived social norms regarding when it was appropriate to stand or sit to be a barrier to changing. These participants thought it would be awkward or inappropriate to stand while everyone else was sitting, particularly in a professional setting. P1510 described,

“*For professionals, when those meetings are face-to-face, there’s a strong social expectation that the participants will sit down at the table and face each other during the meeting. Getting up and walking around is generally interpreted as being pretty significant, and somewhat aggressive, and somewhat disruptive, and so it’s basically not acceptable social behavior for most contexts, at least in my profession.*” (P1510)

However, when participants challenged this perceived social norm, they found the experience to be much more acceptable than anticipated; in some cases, others joined the participant in standing.

“*It is a little awkward to [stand up] in a restaurant. I’ve done it. And, it’s funny—I don’t think anybody even thought to ask me why I stood up. I stayed standing; I didn’t go marching around the restaurant. But, I don’t think anybody even thought anything about it.*” (P1879)

### Facilitators

Participants identified a number of factors that facilitated reducing sitting.

#### Increased awareness

All participants reported that a greater awareness of their own sitting was the most powerful facilitator of change. As P1175 said, “*I was kind of appalled in the beginning to find out how little I stood. I was way off in my estimate of how much I sat.*” Other participants described awareness of sedentary behavior as a “*wakeup call*” (P1133), and “*eye-opening*” (P1053). P1360 said,

“*It was shocking that I sat for over 12 hours a day.… It’s just not something that I thought of before. I mean, I was aware that I sat a lot, but I hadn’t really thought of developing strategies to not sit so much—actively trying to change that behavior. It hadn’t occurred to me.*” (P1360)

Most commonly, participants reported that the activPAL-generated feedback charts were the biggest contributors of increased awareness of sedentary behavior.

“*Getting the measurement so that I could tell what type of day, what day of the week, specifically, I was more active or less active. That I found very valuable. I think that was really, really important, to get some actual numbers on how much I’m sitting, how much I’m standing, and what time of day that is*.” (P1510)

Participants reported that the I-STAND study not only increased their awareness of how much time they personally spent sitting, but of the consequences of sitting.

“*The biggest change I noticed was that the longer I sit, the more apt I am to have pain when I do rise. Which is counter-intuitive because you think sitting helps you, and that’s a mistake. So I find that I get up a little more often—or I think so—spontaneously, just because I know it’ll make me feel better.*” (P1175)

The I-STAND study also helped increase participant awareness of easy ways to reduce sitting. “*I became much, much more aware of how much I sit. I discovered tools to assist me in standing more and monitoring that independently*” (P1188). Participants relied on reminders to increase awareness of sedentary time and to remember to take breaks.

#### Reminders

The I-STAND study taught participants to use various reminder strategies (outward, inner, and habit-associated) to reduce sedentary behavior and take frequent breaks from sitting. Almost all participants used at least one reminder and most used at least two. Outward reminders, which included the Jawbone UP band and other types of alarms or visual signs, were most commonly used at the beginning of the study. Participants reported that they helped remind participants how long they had been sitting.

“*The timer was helpful because I have no innate concept of time. I mean, sometimes, I’d think I’d been standing for a long time, and I look down, and it would be only five minutes. Having something that kept me honest, so to speak, and told me where I was was really helpful.*” (P1697)

Participants also described how outward reminders made them more aware that they had a choice to sit or stand—that sitting was not the only option. P1065 described the visual cue cards he used to remind himself to think twice before automatically sitting: “*…if I put it* [the cue card] *on the chair, I had to move it before I would sit down and, you know, brought back, you know, consciousness I have to stand up.*” Some participants thought the usefulness of outward reminders wore off toward the end of the program, and then relied more heavily on other types of reminders.

“*Then, also, working with my coach, start slowly working more internal triggers for me to key on. It got to where the point was I would get up on my own, and then the Jawbone would buzz me. I was starting to use internal and external cues for 15 or 20-minute sitting periods instead of just depending on the Jawbone like I did at the start*” (P1538)

Inner reminders included a sense of mindfulness or cues from one’s own body (pain, tingling/numbing, or discomfort from prolonged sitting) that reminded participants to take a break from sitting. Towards the end of the study, participants began to rely more on inner reminders as they developed an internal awareness of their sitting.

“*It’s my body talking to me and telling me, but until I had the Jawbone to begin the process, I wasn’t getting it. Okay, so I was uncomfortable and I knew I was uncomfortable but I didn’t have a process. So going through the I-STAND program and having the Jawbone in the beginning helped me develop the body awareness so that I could then listen to my body tell me, ‘You’re uncomfortable. Get up.’*” (P1042)

As P1042 described, participants began to rely less on outward reminders as they became more comfortable using inner reminders. Although not all participants used inner reminders, those who did perceived them to be the most important for sustaining sedentary behavior change.

*“… I just find almost spontaneously, something like, ‘God, I’m not comfortable. I’ve been sitting here too long,’ and I’ll just get up and walk around a little bit. I think it really instilled some sort of more instinctiveness in wanting to feel the movement, so to speak*.” (P1697)

Habit-associated reminders involved pairing daily behavioral habits with standing breaks. For instance, participants would add periods of standing to daily habits like drinking coffee, watching TV, or talking on the phone. Of the three reminder types, habit-associated reminders were the easiest to incorporate into daily routine and seemed to resonate most strongly with participants. P1163 said,

“*I started getting in the habit, like when I’m on the telephone, standing up. And when I’m watching TV, stand up, and during the commercials, I started moving around. I wasn’t just sitting there waiting for the show to come back. And I started getting off of the bus a little bit earlier than just the stop near where I lived or where I worked.*” (P1163)

#### Accountability

Participants reported that accountability played an important role in changing sedentary behavior. Accountability came from biweekly phone calls with an I-STAND health coach, activity monitoring from the activPAL, and personal goal-setting.

“*I think the fact that I had to commit and had somebody actually looking over my shoulder. There was an actual record being kept of when I was sitting and when I wasn’t. So it was something that I knew was being actually measured versus just an estimation on my part.*” (P1053)

#### Daily activities

Many participants thought that staying busy facilitated changes in sedentary behavior and often spaced out activities and chores throughout the day.

“*I think I’m more willing to accept that it’s important to get up, and to try to time activities so that I’m standing more, and doing chores. Instead of bunching them all together where I have to go out and walk around, I do them separately because it means I spend more time out, up, and around.*” (P1304)

Participants also looked for more activities that would keep them on their feet.

“*I found myself looking for things to do during my breaks, or looking for things to do that needed to get done, little things like go do your laundry, or clean the house, or wash the car, mow the lawn.*” (P1578)

#### Social support and norms

Although the I-STAND study focused on individual changes, participants frequently described the positive role other people played in facilitating changes in sedentary behavior. Participants felt encouraged by family, friends, and the I-STAND health coaches to make changes. According to P1538,

“*I think one of the best supporters is that friend of mine who is fit. We used to do stuff together. Now, he’s off doing stuff that I don’t feel I could really join in because I’m not in good enough shape, so I wanna do some of that again. He’s gonna be a good support guy to check in and gently prod me in the right direction.*” (P1538)

P1038 described another way she felt supported:

“*People know that I’m doing this now so I get these strange kitchen timers from people. So I have one for every day of the week… My son actually had gone to Europe on a project and when he was coming back at some airport, he picked me up a very funny timer.*” (P1038)

Participants also described how they looped family and friends into their standing/walking routines.

“*It was a quilting group, and there were nine women involved. And, the day that I got there… they all wanted to know, of course, what the Jawbone was all about. Well, I told them. And so, they all said, “Well, we’ll do it too!” And, all day, every time the Jawbone buzzed, all nine of us stood up. And, we got to the point where we would stand up, and we would do some stretching exercises, or we would just walk around in a little circle, or we would just wiggle, and what not, and sit back down. But, it got to the point where all I had to do was go, “It’s time!” and all nine butts came off the chair.*” (P1879)

Although social norms to sit in certain social settings were a perceived barrier for some, others remarked on the relative lack of judgment they felt in social settings when they would stand.

“*I go to jazz house concerts, and generally speaking I choose the chair in the back where I can stand next to the chair, so a chair on the edge, not in the middle of the row, where I can stand up and listen to the music standing up or sitting down, so not just sitting down. The person who organizes these house concerts has now started putting stickies on those chairs with my name on it, so it’s become official.*” (P1133)

Finally, participants sometimes noticed that they became more social when they reduced their sedentary time, which, in turn, increased their interest in further reducing their sitting.

“*And, actually, I don’t mind walking around the floor at work because sometimes I can stop and say hi to people I normally don’t see. You know, they’re not on the same hallway system. So I probably will continue to do that at least sometimes.*” (P1163)

#### Home environment

While intervention content focused on changing the home environment to facilitate taking breaks from sitting, it did not emerge as a strong barrier or facilitator. Most participants remarked that they could not make adjustments to their home because they had a small living space or resided in assisted living. However, participants did describe changes in the way they interacted with their home environment. P1188 remarked, “*I had to work around my furniture—so the environmental things did not change, what I did around them changed.*” Several participants described using the back of tall chairs or handrails as supports to lean against, and several repurposed kitchen counters and other tall surfaces into standing desks for computer work or crafts. “*I discovered with this program that if I use my standing area in the kitchen at the counter, I have a designated space for me to do my work standing, and I will do it*” (P1042). The environment was previously found to have an important impact on sedentary behavior [18], so it is surprising that only about half of participants in this study discussed the home environment as a facilitator for reducing sedentary time and no participants described it as a barrier to behavior change. Other environmental factors such as the weather, long commutes, and office space were infrequently mentioned.

### Perceived health impact

All participants perceived that participating in the I-STAND intervention had some positive impact on their health. Participants described varying benefits they experienced from reducing sedentary time.

#### Physical benefits

Most commonly, participants attributed improvements in physical health to changes in sedentary behavior. Over the course of 12 weeks, participants said they lost weight and saw improvements in other chronic conditions (e.g., lower blood pressure, resolution of sciatica, less pain). P1038 said, “*I’m losing a couple of pounds and have a little more energy.*” P1199 added, “*I have kind of a 30-years chronic lower back problem that I think has improved as a result of doing this… I think my core muscles are a little bit stronger as a result of standing more frequently.*” Participants also noted increased flexibility, muscle strength, and a greater ability to get up and move.

“*I can tell my strength, just my overall body strength, has improved, and I feel my balance is better, sorta feel that my legs are stronger, and just I’m less winded when I go up a set of steps.*” (P1538)

#### Increased energy

Participants also remarked that reducing their sedentary time increased their energy. Taking more frequent breaks from sitting and going on walks warded off feelings of sluggishness: “*Yes, because I think, the more you sit, you get sluggish. So, yes, the more I sit, it does negatively impact my energy level*” (P1879). Participants also commented on the reinforcing relationship between standing/walking and energy.

“*I feel I have more energy, so it’s sort of a positive feedback loop. Because of doing this, I have more energy, so I’m taking care of chores better. Of course, taking care of chores gets you up moving around more and less sitting, so it’s a—instead of a downward cycle, the I-STAND program changed it to an upward cycle.*” (P1538)

#### Increased interest in physical activity

Although physical activity was not specifically a goal of the I-STAND study, participants said that after completing the I-STAND study, they felt more prepared to do physical activity.

“*My body feels like it’s in a better position to start incorporating more walking and maybe some aerobic stuff, whereas before I would do that, it was not quite painful, but not pleasant. Now, it feels good again to walk, so that was a good thing.*” (P1538)

For participants like P1538, having a goal to reduce sedentary behavior was a first step towards increasing physical activity. Other participants felt that physical activity goals complemented their sedentary goals and looked for ways to incorporate both decreasing sedentary time and increasing physical activity into their daily routine.

“*I had gotten to a point where I had screwed things up so much that I was having a hard time doing—I used to do five-mile walks every morning and arthritic knees and tearing things in your knees, it was just not as easy to do anymore. But I discovered by doing five-minute walks every 25 minutes, I get my 10,000 steps in in a day and my knees don’t mind. So that little push to be more aware of my sitting made some differences that way.*” (P1038)

#### Improved mood

Some participants correlated improved mood with reduced sedentary behavior. P1163 said,

“*I was really depressed the couple of months before the study started. And all of a sudden I’m concentrating on something different and I’m getting out and trying to conscientiously do these extra things like standing and walking because I wanted to get the steps in. And I just think mentally I’ve been feeling a lot better.*” (P1163)

Participants frequently attributed this boost to better utilizing their time and engaging in more activities that they enjoy (e.g., gardening, walking, crafting, socializing, etc.). One participant commented that the I-STAND study changed her mind set about sedentary behavior and helped her feel more positive about her role as a caretaker, which can involve frequent breaks from sitting.

“*Well, part of it is, as much as I love my father, and I’m happy to have him here and care for him, it can be annoying when I just sit down and feel like, oh, I can relax, and he calls for me. So now I know that that’s a good thing. Getting up is good, going to him is good; it made me not view it as this interruption. That it was a good thing. And then I felt like I was doing something to help myself. I think that made me more positive.*” (P1175)

Some participants also attributed improved mood with a sense of achievement and a greater confidence in their ability to reduce their sitting. “*Yes, it puts me in a very nice, happy mood. But it can be just, you know, myself. I feel really good about it, accomplishing something, knowing that I’m actively standing and doing this standing really helps*” (P1065).

#### Reduced stress

Participants did not report any additional stress from participating in I-STAND or from efforts to reduce sedentary behavior. In contrast, some participants added that reducing their sitting time as part of the I-STAND program may have actually reduced stress because they were able to accomplish more activities during the day. “*The only thing is by taking care of a situation as they arise, it alleviates any of the stress of knowing that there’s something there that still needs to be done*” (P1053). Other participants said that they felt less stress by increasing physical activity. “*I have been getting off the bus a couple of stops earlier at the end of the day… And it gives me time just to kind of, what is it, de-stress before I get in the house*” (P1163).

## Discussion

We sought to determine the acceptability of a sedentary behavior intervention for older, obese adults at high risk of chronic diseases, to describe barriers and facilitators to reducing sedentary behavior, and to understand the perceived health impact of reduced sedentary behavior. This study also had the opportunity to assess older adults’ comfort with and interest in using technology to facilitate sedentary behavior change.

Overall, the I-STAND intervention was highly acceptable among older adults with obesity. I-STAND participants felt they were able to reduce their sedentary behavior, and they were satisfied with intervention components that helped them do so. Participants found it easy to incorporate I-STAND practices and program components into daily life. Participants did not necessarily use every program component available to them but worked with their health coach to select strategies that fit their lifestyle and preferences. Most reported that habit reminder strategies, such as standing for 5 min after finishing each section of the newspaper, were helpful. Many also used outward reminders, such as the Jawbone UP band or cue cards, to facilitate sitting reduction, and some found inner reminders, such as body awareness, to be strong reminders, particularly toward the end of the intervention. Although most participants described positive experiences, participants also offered suggestions to improve I-STAND program components, specifically related to making technology age-appropriate. Older adults are using technology more than ever before [25, 26]; however, previous qualitative findings have suggested that technology should not be included as a solution to reduce sedentary behavior in older adults [17]. Our results partially contradict this claim; older adults in the I-STAND intervention were interested in using technology (e.g., wearable devices, smart phone apps, web portal) as long as the technology is easy to use and does not replace personal interactions with a health coach. Qualitative feedback suggests technology should be tailored to detect older adult movements, which may be different from younger adults [27], and designed to accommodate those with limited vision, hearing, and nimbleness. Our findings also highlight the need to provide a menu of program components (with and without technology) so that participants may choose the components that are most appropriate and helpful for them.

Barriers to reducing sedentary behavior included poor health, ingrained sedentary habits, preference for sedentary behavior, and perceived social norms about sitting, while facilitators included increased awareness of sedentary behavior, a sense of personal accountability, daily activities, social support, and changing ways of interacting in the home environment. Barriers were consistent with previously identified barriers [18]. Poor health (including pain) and a preference for sedentary behavior are also common barriers to participating in physical activity [28], but neighborhood environmental barriers, which are regularly cited in physical activity interventions [29], were less frequently mentioned in this study. While prior studies have used standing desks in worksites with evidence of efficacy [30], most interview participants did not modify their environment. Instead, participants learned adaptive ways to stand and move in their home or office. This may be a more feasible strategy for older adults who have less control over their environment. Ingrained sedentary habits and perceived social norms are unique barriers to sedentary behavior change and highlight the need for greater cultural awareness about sedentary behavior. Increased awareness was one of the most frequently cited facilitators to reducing sedentary behavior. Participants felt strongly about incorporating activity into their daily routine that would break up periods of prolonged inactivity. The strength of ingrained, automatic sedentary habits and the important role of increased awareness paired with continual reminders to take breaks from sitting underscore the habit formation dynamics underlying sedentary behavior. Habit formation theory posits that unconscious decisions underlie sitting behavior [20]. Disrupting these automatic habits and bringing them into conscious awareness with frequent cues helped promote decisions to stand and move more frequently. Over time, these cues may not be required as the decision to stand instead of sit becomes more automatic and habitual.

All participants reported that sitting less resulted in health benefits such as improved physical health, increased energy, greater interest and confidence in engaging in physical activity, improved mood, and reduced stress. These findings are consistent with previous qualitative findings [18] although reduced stress emerged as a new perceived benefit. Participants suggested this reduced stress was a result of doing more activities that gave meaning to their lives (e.g., gardening, exercise, socializing) and was related to improved mood. Participants did not report changes in mental health, possibly because self-reported anxiety and depression were low at baseline. Future studies can determine whether these perceived health benefits are consistent with objective measures of improved health.

The validity and generalizability of these findings must be interpreted in the context of potential limitations. Like all qualitative studies, the findings may be biased by the researchers. However, our team-based, iterative approach to analyzing the data improved the rigor of our investigation. Retrospective accounts may have also limited participant ability to recall details of their experience. To minimize limitations to recall, participants were interviewed by phone within 10 days of their last follow-up visit. Social desirability bias may have resulted if participants felt compelled to describe their experience in the I-STAND study favorably. We attempted to reduce any perceived pressure by informing participants at the beginning of each interview that the study staff would not know the name of interview participants, that all feedback—even negative feedback—was valuable to improving the study, and that participants were free to skip any question that they did not feel comfortable answering. Further, participants were not offered additional incentives for completing the exit interviews. Finally, we facilitated honest feedback by using a neutral study team member who had no prior contact with participants to conduct the interviews. Generalizability of the results is limited by the use of a convenience sample of KPWA patients who agreed to participate in the I-STAND study. Participants who agreed to be interviewed may not be representative of the general population of older adults. A strength of this study, however, is our recruitment of older adults at risk of chronic diseases as these individuals may benefit the most from a sedentary reduction intervention and are often excluded from research studies. Future studies should attempt to recruit a more diverse sample to see if qualitative experiences are shared across other populations. This study is also strengthened by the length of the intervention (12 weeks) during which time participants were able to observe changes in their sedentary habits and health. Quantitative findings help validate these changes.

## Conclusion

Two previous qualitative studies have asked older adults how they would feel about reducing their sedentary behavior [17, 19], but only one other study assessed the perceived impact of sitting less on older adults [18]. This study expands on prior research by interviewing older adults with experience making sedentary behavior changes over a prolonged period; it is also the first qualitative study to evaluate the use of technology in a sedentary behavior intervention with older adults. Because there are so many misconceptions about sedentary behavior [5], these post-intervention, patient-centered assessments are important for understanding the feasibility [18], acceptance, and perceived impact reducing sedentary behavior has on older bodies. An overall positive perceived health impact of sitting less helps to fill important gaps in the literature. Further, participant feedback about the I-STAND intervention, the technology, and other program components will help future researchers design sedentary behavior interventions that meet the needs of older adults at risk for chronic diseases. Taken together, this study presents early qualitative results that suggest a technology-enhanced sedentary reduction intervention is acceptable, easy to incorporate, and has a positive perceived health impact on older adults at risk of developing chronic diseases.

## Additional files

Additional file 1: I-STAND Semi-Structured Exit Interview Guide. (DOCX 18 kb)

Additional file 2: Selected quotes illustrating acceptability of I-STAND program components, barriers and facilitators to achieving sitting reduction goals, and perceived health impact of reducing sedentary time. (DOCX 26 kb)

## Abbreviations

BMI: Body mass index
I-STAND: Investigating Sedentary Time in Aging: New Directions using technology
KPWA: Kaiser Permanente Washington
MET: Metabolic equivalent task
