# Supplementary material for: Acceptability of a sitting reduction intervention for older adults with obesity
Source: BMC Public Health. 2018 Jun 7;18:706. doi: 10.1186/s12889-018-5616-1 (PMC5992825; doi:10.1186/s12889-018-5616-1)
Supplement: Supplementary file 1 — I-STAND Semi-Structured Exit Interview Guide. (DOCX 18 kb) [file 12889_2018_5616_MOESM1_ESM.docx]

**Additional File 1.** I-STAND Semi-Structured Exit Interview Guide

| **DOMAIN** | **QUESTION** | **PROBES** |
| --- | --- | --- |
| 1. **Program overall** | What did you think of the ISTAND program overall? | - What did you think of the changes in your sedentary time from participating in the program? - What helped you meet your sitting reduction goals? What parts of the intervention were the most helpful? (Prompts: in-person sessions, phone calls, goal-setting, feedback charts, workbook, Jawbone, etc.) - What made it difficult to meet your goals? What parts of the intervention were least helpful? - How did your perceptions or attitudes towards your own sedentary time change while you were in the study? - How did the I-STAND program fit/not fit into your daily life? - The study focused on reducing your sitting by one hour per day. You worked on doing more periods of standing and taking frequent breaks from sitting. - Were you able to take more breaks from sitting? If yes, what helped you? If no, what made it hard to? - Were you able to add more periods of standing time into your day? If yes, what helped you? If no, what made it hard to? - How did the program impact your physical activity? - You worked on 3 types of reminders: inner, outward, and habit reminders.   - Did these 3 types of reminders make sense to you?   - What types of reminders were most helpful for you? Why? - How did you make changes to your home environment? How about other places where you spend time (e.g. work)? |
| 1. **Health coach sessions** | What are your thoughts on your experiences regarding your health coach sessions? | - What did you think about the number of sessions (2 in-person and 4 calls)? - What did you think about the format? Would you have preferred another type of format? (Prompts: email based, web-based, smart phone application) - What did you really like/dislike about the health coach sessions? - What could be done to make the sessions more effective? - How would you have engaged in the program if we gave you one health coach session in-person and the rest on a website or smartphone application? - What are the top 2 or 3 most important or helpful parts of I-STAND that we cannot do without? |
| 1. **Technology** | What did you think of the Jawbone device? | - How did you use it? What was helpful/not helpful? - What did you like/not like about the Jawbone? - The Jawbone also syncs to a smart phone application and can provide you with more information on your steps and activity level. Would you have wanted access to that? - What other technologies would have been helpful to have? |
| 1. **Feedback charts** | What did you think of the feedback charts you received? | - What was helpful/not helpful? - Was it worthwhile to wear the activPAL for 4 weeks during the study so you could get the feedback charts? Why? - I noticed from your final feedback chart that you improved (fill in improved metrics), what do you think led to your success? - I noticed from your final feedback chart that it looked like it was harder to (fill in metrics they did not improve), what made it difficult to target (fill in metric name)? |
| 1. **Health benefits** | Did you notice any changes in your health as a result from participating in this study (physical or mental)? | - How did you feel on days you sat less? - What changes, if any, have you noticed in your mood? (Prompt: depression, anxiety, cognition, ability to focus, energy level, etc.) - What changes have you noticed in your body? (Prompt: stiffness, pain, fatigue or ability to do daily activities) |
| 1. **Conclusion** |  | - What do you think will happen now that the study has concluded? What strategies do you plan on continuing to use? - Can you tell me about what made you volunteer for the study? - Is there any other feedback you would like us to have? - Are there any questions I can answer for you? |
