# Supplementary material for: Acceptability of a sitting reduction intervention for older adults with obesity
Source: BMC Public Health. 2018 Jun 7;18:706. doi: 10.1186/s12889-018-5616-1 (PMC5992825; doi:10.1186/s12889-018-5616-1)
Supplement: Supplementary file 2 — Selected quotes illustrating acceptability of I-STAND program components, barriers and facilitators to achieving sitting reduction goals, and perceived health impact of reducing sedentary time. (DOCX 26 kb) [file 12889_2018_5616_MOESM2_ESM.docx]

**Additional File 2.** Selected quotes illustrating acceptability of I-STAND program components, barriers and facilitators to achieving sitting reduction goals, and perceived health impact of reducing sedentary time.

| **Acceptability of sedentary behavior reduction intervention** | |
| --- | --- |
| Overall satisfaction | Overall, I thought the program was good. I liked my coaches. I liked the material that was issued. I think the manual helped a lot. – P1284  I really like it. I was kind of impressed. I wasn’t sure when I first signed up, but I found it very helpful. Everybody that I dealt with was very supportive and pleasant to deal with. I think it was a very worthwhile experience for me. – P1697  I liked the fact that there was a coach to it, and that there was a notebook to follow, and various suggestions about what to do, and how to overcome barriers and things like that. - P1304  I thought it was interesting and well worth my time. - P1697  I think having a [health coach who] helped me with the goal setting was incredibly valuable. Not just the goal setting but the follow up of the goal setting and the future planning for at the end of the study—what am I gonna do then. – P1078  It’s just been a wonderful experience, and it’s changed my life, and I can’t say more than that. That’s what you guys wanna do. - P1042 |
| Technology (activPal and Jawbone) | [The **activPal**] was very helpful. And it wasn't intrusive; you forgot it was on, right. It was tiny enough. – P1163  [The **activPals**] were probably one of the best resources to monitor my daily movements, and I thought they were fabulous – P1188  I don’t know why [the **activPal**] bothered me—having that on my leg. Especially that first one. But you know, they work and I can understand why they need it to be there. – P1065  I think I might have liked wearing that [**activPal**] a little more often. I kind of liked that, and I thought that was a great device to get you to do the things that you know you have to do because it’s being documented and you want the results to be positive. So it’s kind of hard to cheat when you have that thing strapped to your leg. – P1578  The little buzz [the **Jawbone**] would give you was very helpful in a way that, say, just trying to keep track of time on your own is not. – P1255  That **Jawbone** was probably the best reminder of everything during the full course of the program. – P1538  I would have very willingly wore a [**Jawbone**] if it was more easy, you found one that is not dark, and one that’s easy to see, and a wristband that the patient or a participant can manipulate – P1110  [The **Jawbone**] did not appear to vibrate at the right times. In other words, when I was being active, it would vibrate. That didn’t make any sense, so after a while, I basically decided its input wasn’t helpful. – P1510  Let's see, so at the beginning [the **Jawbone**] was a good reminder that I should stop sitting and stand up. Towards the end, it became more ingrained in my brain, so I didn't need the **Jawbone** as much. – P1133 |
| Ease and effectiveness | It took a lot less effort to actually accommodate the program than what I thought it would. – P1053  It was definitely feasible to work the strategies in with my daily work and my recreation, yeah. – P1510  I surprised myself how absolutely sedentary I was, and then how I was able to make some changes that actually didn’t feel really difficult. I was thinking, “Oh, my god, I’m gonna have to stand here,” but it wasn’t like that. Anyway, it was a positive experience.… It fit in quite easily. I mean, it didn’t feel like, “Oh, I have to do this.” It worked quite well in the sense that it didn’t feel as it was some sort of effort. – P1697  You don’t need special equipment, you don’t have to have the right kind of clothes, you don’t have to go anyplace, it doesn’t cost you a gym membership. You just have to stand up. – P1879  And I’ve noticed in the three days since the study ended, I’m still getting up when I’m watching TV and things like that—getting up and stretching and walking around. – P1199  But now, it's just a part of the day. You don't think about it anymore. It's just your routine. – P1038 |
| **Barriers to reducing sedentary behavior** | |
| Poor health | If I’m feeling tired, it’s much harder for me to get up and move around. – P1169  Well, I fell. Landed flat on my face and didn’t feel too great. So there was a period there. And I’ve been running a fever for almost a week, so I’ve just kind of felt puny. – P1175  I was changing some blood pressure medication, and they were causing a lot of fatigue, and I think that that changed my activity as well. – P1199  Well, I’m a heavy woman, and so it’s hard to stand in one place because it just—it makes my knees hurt. It’s just uncomfortable. – P1188  I also have a physical problem. I thought if I stand for more than half hour at a time, my back problems, my spine problems are very problematic because they bear a lot of discomfort – P1110 |
| Ingrained sedentary habits | The habit of always sitting no matter where I was or what I was doing is, for the most part, is I’d just gotten into the habit of sitting, whether it was watching TV, reading, or whatever. And that was about it. That was about the worst. I was trying to overcome that. – P1053  I went to the bus stop, and we have a bench at the bus stop. My friends were seated, and I automatically joined them, and then I felt guilty and jumped right back up again. So it is weird. – P1163  So you know, a lot of your activity is sitting, so it’s a challenge. You have to kind of think about it, which I didn’t really do much before; thinking about whether I was standing or sitting. – P1360  So it was a little bit of a challenge to get into that—to break that habit of not getting up. And if you’re watching a movie or something, if it’s not something that’s not interrupted continually with commercials, it’s a little hard to put it on pause and go walk around for a couple minutes. – P1578  I think the older you get, the longer it takes [to break sedentary habits]. – P1879 |
| Lack of motivation and competing priorities | I’m old and lazy, and I have pain sometimes and so then I don’t do what I know I should. And **creature** comforts become more important as you get older. - P1042  To tell the truth, most of the things that I enjoy doing most are sedentary activities. We watch TV in the evening, and my favorite hobbies during the day are reading and knitting, both of which are sedentary, generally sedentary activities. – P1255  I was looking forward to this aspect of retirement that I, you know—I’d be able to indulge some of my other passions like reading, studying, intellectual pursuits… And I kind of resented this idea of: What do you mean, I can’t sit down*?* – P1086  Well, I have a son who had to have surgery, and so there were just some stresses in my life that prevented me from—I wasn’t really paying attention to my goals at that time. – P1637  I've got caretaking responsibilities, and they come before standing up if I'm in the middle of doing something that requires me to sit down with the baby. – P1133 |
| Social norms | For professionals, when those meetings are face-to-face, there’s a strong social expectation that the participants will sit down at the table and face each other during the meeting. Getting up and walking around is generally interpreted as being pretty significant, and somewhat aggressive, and somewhat disruptive, and so it’s basically not acceptable social behavior for most contexts, at least in my profession. – P1510  I have a weekly staff meeting, and so when the Jawbone would go off, I would tend to ignore it because it just didn’t seem like an appropriate thing to do, I guess. – P1637  I can’t stand when I'm out to dinner or a lot of social occasions. There are some bars where you can stand and some restaurants that have standing tables, but they’re not common. But I think about it now, where I didn’t before. So it’s on my mind all the time.… We had a dinner party the other night, and I sat for awhile and then I got up and started clearing the table as a way to not just keep sitting. That’s one way I'm thinking about ways that I can get up during a social situation and move around. – P1360  It is a little awkward to [stand up] in a restaurant. I’ve done it. And, it’s funny–I don’t think anybody even thought to ask me why I stood up. I stayed standing; I didn’t go marching around the restaurant. But, I don’t think anybody even thought anything about it. – P1879 |
| **Facilitators to reducing sedentary behavior** | |
| Increased awareness | I was kind of appalled in the beginning to find out how little I stood. I was way off in my estimate of how much I sat. – P1175  I would say the first part sort of making you aware of this is the percentage of time you're spending sitting, lying down, standing, walking. It's quite a wake-up call, right? – P1133  It was shocking that I sat for over 12 hours a day.… It’s just not something that I thought of before. I mean, I was aware that I sat a lot, but I hadn’t really thought of developing strategies to not sit so much–actively trying to change that behavior. It hadn’t occurred to me. – P1360  I became much, much more aware of how much I sit. I discovered tools to assist me in standing more and monitoring that independently. – P1188  So I think my awareness is a lot better, and there’s an immediate feedback loop of feeling—it’s just that feeling you have when you stretch your arms or you stretch your legs; it just feels good for a moment. So there’s that feedback loop that rewards it. Now that I know that I can do that, I do it. And for me, the biggest issue was remembering to do it. – P1199  It makes you aware of how much time is being wasted just sitting and not moving and how much it affects your overall mood and your wellbeing – P1053 |
| Accountability | I think the fact that I had to commit and had somebody actually looking over my shoulder. There was an actual record being kept of when I was sitting and when I wasn’t. So it was something that I knew was being actually measured versus just an estimation on my part. – P1053  It’s always good when you have to face somebody, and you made promises about goals you’re gonna keep, and you haven’t done that. The first time, maybe if you screw up the first week, then by the second week, you’re like, “Oh,” you pay more attention to it. – P1538  I think that the measuring device was significant, the phone calls. Having some accountability built into the study was good. – P1637  I really appreciated the ongoing visits on the phone so that it kept me on my toes. – P1163 |
| Daily activities | I think I’m more willing to accept that it’s important to get up, and to try to time activities so that I’m standing more, and doing chores. Instead of bunching them all together where I have to go out and walk around, I do them separately because it means I spend more time out, up, and around. – P1304  I found myself looking for things to do during my breaks, or looking for things to do that needed to get done, little things like go do your laundry, or clean the house, or wash the car, mow the lawn. If you use your breaks to do those kinds of things, then you look for things to do rather than have things come and looking for you to get done. It seems to make life easier. – P1578  I looked for projects to do that would keep me occupied over the sitting and just busy work. – P1053  I tried to inconvenience myself and become very inefficient. It's like when I do laundry, instead of taking the basket into whatever room I'm going to put the things in, I take everything into the living room and then – it's a silly game, but I will take one towel at a time and walk it into the room where the towels go, one pair of socks and walk it into the bedroom. So I get a ton of steps in when I do the laundry just because I play that game of don't make it convenience and efficient. Try to be as inefficient as you can. – P1038  If I use my standing area in the kitchen at the counter—we have an island also that has a lot of space—now I have a designated space for me to do my work standing, and I will do it. – P1042 |
| Social support and social norms | I think one of the best supporters is that friend of mine who is fit. We used to do stuff together. Now, he’s off doing stuff that I don’t feel I could really join in because I’m not in good enough shape, so I wanna do some of that again. He’s gonna be a good support guy to check in and gently prod me in the right direction. – P1538  People know that I'm doing this now so I get these strange kitchen timers from people. So I have one for every day of the week… My son actually had gone to Europe on a project and when he was coming back at some airport, he picked me up a very funny timer. – P1038  It was a quilting group, and there were nine women involved. And, the day that I got there… they all wanted to know, of course, what the Jawbone was all about. Well, I told them. And so, they all said, “Well, we’ll do it too!” And, all day, every time the Jawbone buzzed, all nine of us stood up. And, we got to the point where we would stand up, and we would do some stretching exercises, or we would just walk around in a little circle, or we would just wiggle, and what not, and sit back down. But, it got to the point where all I had to do was go, “It’s time!” and all nine butts came off the chair. – P1879  And, actually, I don’t mind walking around the floor at work because sometime I can stop and say hi to people I normally don’t see. You know, they’re not on the same hallway system. So I probably will continue to do that at least sometimes. – P1163  When I’m out in public, I don’t seem to have as much problems sitting for great lengths of time, but if I have been in some meeting or something where I feel the need to stand up—they’ll give you a break once every hour and a half—I don’t hesitate at all to stand up and go stand in the back of the room. – P1188  When I’m with a group of friends—if they’re close friends—they don’t care whether I stand up. That doesn’t bother them at all. – P1879 |
| Home environment | I had to work around my furniture—so the environmental things did not change; what I did around them changed. – P1188  I realized I could take one of my kitchen chairs and put it in the living room so I can stand while I’m watching TV and just have something to lean on a little bit. – P1538  I discovered that the hand railing around our deck is just a perfect leaning height, so I stand at my deck and read a lot. – P1199  I discovered with this program that if I use my standing area in the kitchen at the counter, I have a designated space for me to do my work standing, and I will do it. – P1042  Sometimes I’ve taken some easy chairs and put pillows on them or something, and I—so, if I’m going to sit down there, I’d have to pick the pillows up. – P1086 |
| **Perceived health impacts** | |
| Physical benefits | I noticed that my stamina is a lot better. I used to huff and puff and now I feel just better so I really liked it. – P1163  I'm losing a couple of pounds and have a little more energy. – P1199  I have kind of a 30-years chronic lower back problem that I think has improved as a result of doing this… I think my core muscles are a little bit stronger as a result of standing more frequently. – P1038  I can tell my strength, just my overall body strength, has improved, and I feel my balance is better, sorta feel that my legs are stronger, and just I’m less winded when I go up a set of steps. – 1538  I’m not having the swelling of my ankles like I did before. It was really bad. I was quite uncomfortable. – P1042  I’ve been on blood pressure medicine for a couple of years, and I’ve been trying to work to get off that, and I’ve now got my blood pressure down low enough where I no longer have to take the blood pressure medicine, so I think the I-STAND program helped me out quite a bit there. – P1538 |
| Increased energy | Yes, because I think, the more you sit, you get sluggish. So, yes, the more I sit, it does negatively impact my energy level. – P1879  I feel I have more energy, so it’s sort of a positive feedback loop. Because of doing this, I have more energy, so I’m taking care of chores better. Of course, taking care of chores gets you up moving around more and less sitting, so it’s a—instead of a downward cycle, the I-STAND program changed it to an upward cycle. – P1538  I have the energy to get out there and not only pull that grass but break the dirt and make sure all of it is gone that I haven’t done before ever. So and we’ve been here ten years. – P1042  I think it helps you concentrate. You know… the blood’s not all pooling in your lower extremities. You’re able to think more clearly and more consistently and not drift off into daydreaming or something like that. – P1086 |
| Increased interest in physical activity | It has actually given me more of a boost to get back into exercising as well. – P1255  I used to do five-mile walks every morning and arthritic knees and tearing things in your knees, it was just not as easy to do anymore, but I discovered by doing five-minute walks every 25 minutes, I get my 10,000 steps in in a day and my knees don't mind. – P1038  My body feels like it’s in a better position to start incorporating more walking and maybe some aerobic stuff, whereas before I would do that, it was not quite painful, but not pleasant. Now, it feels good again to walk, so that was a good thing. – P1538  I think I snuck in a little bit of bike exercise. Although it’s a recumbent bike, I am moving my legs a lot. That was a little bit more activity. I would walk around more, as I say, more excuses to go out and, say, go to the library in one trip, and go to the drugstore in another, and stuff like that. – P1304 |
| Improved mood | I was really depressed the couple of months before the study started. And all of a sudden, like I'm concentrating on something different, and I'm getting out and trying to conscientiously do these extra things like standing and walking because I wanted to get the steps in. And I just think mentally I’ve been feeling a lot better. – P1163  “Well, part of it is, as much as I love my father, and I’m happy to have him here and care for him, it can be annoying when I just sit down and feel like, oh, I can relax, and he calls for me. So now I know that that’s a good thing. Getting up is good, going to him is good; it made me not view it as this interruption. That it was a good thing. And then I felt like I was doing something to help myself. I think that made me more positive. – P1175  Yes, it puts me in a very nice, happy mood. But it can be just, you know, myself. I feel really good about it, accomplishing something, knowing that I'm actively standing and doing this standing really helps. – P1065  I don’t have the low days like I used to have. - P1042 |
| Reduced stress | I think it might have reduced my stress level. – P1510  The only thing is by taking care of a situation as they arise, it alleviates any of the stress of knowing that there’s something there that still needs to be done. – P1053  I have been getting off the bus a couple of stops earlier at the end of the day… And it gives me time just to kind of, what is it, de-stress before I get in the house. – P1163  I think it’s helped me deal with all these big things happening. I’ve had a bunch of emergencies.… But I think just being a little bit more fit has helped me deal with that stress more, and especially the walks. – P1538 |
